# Nanochannel array modified three-dimensional graphene electrode for sensitive electrochemical detection of 2,4,6-trichlorophenol and prochloraz

**DOI:** 10.3389/fchem.2022.954802

**Published:** 2022-09-07

**Authors:** Weiran Zheng, Ruobing Su, Xingyu Lin, Jiyang Liu

**Affiliations:** ^1^ Institute of Agro-product Safety and Nutrition, Zhejiang Academy of Agricultural Sciences, Hangzhou, China; ^2^ Department of Chemistry, Key Laboratory of Surface and Interface Science of Polymer Materials of Zhejiang Province, Zhejiang Sci-Tech University, Hangzhou, China; ^3^ College of Biosystems Engineering and Food Science, Zhejiang University, Hangzhou, China

**Keywords:** electrochemical sensor, three-dimensional graphene, nanochannel array, 2,4,6trichlorophenol, prochloraz

## Abstract

Convenient, and sensitive detection of pesticides and their metabolites in environmental or food samples is critical for assessing potential environmental and health risks. Here, a three-dimensional (3D) electrochemical sensing platform is proposed based on the integration of nanochannel array on pre-activated 3D graphene (p-3DG) electrodes with no need of additional adhesive layers, which enables sensitive detection of prochloraz and 2,4,6-trichlorophenol (TCP) in environmental and food samples. Through two-step electrochemical polarization, organic phase anodic oxidation, and aqueous phase cathodic reduction, p-3DG electrodes with high active area and excellent electrocatalytic performance were obtained. Vertically-ordered mesoporous silica-nanochannel film (VMSF) can be rapidly grown on the surface of p-3DG by an electrochemical-assisted self-assembly (EASA) method. Taking advantage of the high electrocatalytic activity of p-3DG and the ability of nanochannels to enrich TCP through hydrogen bonding, the VMSF/p-3DG sensor can sensitively detect TCP in the range of 10 nM to 0.1 μM and 0.1–15 μM with a low limit of detection (LOD) of 2.4 nM. Compared with p-3DG and VMSF-modified 2D electrodes, the fabricated sensor has a wide detection linear range and low LOD. The coexistence of model interferents such as protein, surfactant, and humic acid did not affect the electrochemical response of TCP, confirming the high anti-fouling ability of the VMSF/p-3DG sensor. In addition, prochloraz in vegetable and fruit samples was indirectly determined because TCP was the metabolite of prochloraz.

## Introduction

The large-scale application of pesticides has led to an increasingly serious problem of pesticide residual pollution in the environment. Prochloraz (PCZ) is an efficient broad-spectrum fungicide. At present, prochloraz has been widely used in the production, storage and transportation of various agricultural and forestry products (Li et al., 2010). For instance, it is applied to treat plant diseases (e.g., oil crops, cereals, tropical and subtropical fruits, vegetables and various economic crops) caused by pathogenic bacteria (e.g., Cercospora, Sclerotinia, Sclerotinia, Sclerotinia, Fusarium and Powdery mildew, Anthrax) (Henry and Sisler, 1984). However, prochloraz has strong biological toxicity including skin irritation, severe disruption of mammalian endocrine levels, etc. Overexposure can also cause serious mutagenic, carcinogenic, or teratogenic effects on mammals (Zheng et al., 2015; Karimipour et al., 2021). In addition, the metabolic product of prochloraz is 2,4,6-trichlorophenol (TCP), which has significant pathological effects and potential carcinogenicity (Li et al., 2010). Due to its great health hazards to humans and animals, as well as its strong chemical stability and refractory degradation, TCP has been designated as one of the environmental priority pollutants by the US Environmental Protection Agency (Buledi et al., 2021; Gopi et al., 2021). Therefore, simple and sensitive detection of prochloraz and TCP in environmental samples are of great significance.

Until now, the quantification of prochloraz is usually achieved indirectly by determining TCP. Traditionally, chromatographic-mass spectrometry or tandem mass spectrometry techniques are commonly used to detect trace levels of TCP (Gopi et al., 2021). Despite good reproducibility and low detection limits, these methods suffer from the requirements of complicated sample pretreatment, high sample consumption, expensive instrument, and professional operators. In comparison with other detection methods (e.g. chromatography or optical detection) (Cui et al., 2020; Deng et al., 2021; Wan et al., 2021), electrochemical sensors have advantages of convenient operation, rapidity, and simple instrumentation (Lu et al., 2018; Yang et al., 2022; Zhou et al., 2022a). As TCP is electrochemically active and can generate electrochemical signals on electrodes, electrochemical detection of TCP and prochloraz is highly desirable. However, due to the interference of co-existing redox molecules and the contamination of large biomolecules and particles, as well as their trace levels in complex samples, the direct electrochemical detection of TCP and prochloraz without tedious pretreatment such as separation is difficult to achieve with common electrodes. Thus, it is highly desirable to develop facile and effective methods to improve the detection sensitivity as well as the anti-interference and anti-fouling abilities of sensing electrodes.

Introducing function nanomaterials to achieve signal amplification is an effective strategy to improve the sensitivity of electrochemical detection. Graphene materials have attracted much attention due to their unique structure, multidimensional scale (e.g., 0D quantum dots (Yan et al., 2019; Cui et al., 2021; Liu et al., 2022), 2D nanosheets (Chang et al., 2022), 3D foam (Chen et al., 2011; Gong et al., 2022a)) and unique physicochemical properties, such as high theoretical specific surface area (∼2600 m^2^g^−1^), excellent electron transport properties, and chemical stability (Wang et al., 2014). However, as single-atom-thick two-dimensional (2D) nanomaterials composed of sp (Henry and Sisler, 1984) hybridized carbon atoms, graphene sheets possess strong π-π interactions and are easy to aggregate and stack. This will significantly reduce active surface area of graphene and the mass transfer of the analyte, thereby reducing the performance of graphene-modified electrodes. Three-dimensional graphene (3DG) prepared by chemical vapor deposition (CVD) using nickel foam as a template has a continuous and seamlessly interconnected graphene monolithic structure with unique macroporous scaffolds and high conductivity (Chen et al., 2011). Thus, 3DG overcomes the agglomeration between graphene sheets, and possesses a large electroactive area, high electrical conductivity, and excellent transport and diffusion of substances (Qiu et al., 2018). However, high hydrophobicity and defect-free structure of 3DG endow it with low wettability and lack of electrocatalytically active sites, thus suppressing its electroanalytical performance. The development of simple methods to modify 3DG to enhance its hydrophilicity and electrocatalytic performance is crucial to expand its application in high-sensitivity electrochemical detection.

Decorating electrodes with vertically ordered mesoporous silica-nanochannel film (VMSF) is an effective way to improve their anti-interference and anti-fouling capabilities (Zhou et al., 2022c; Ma et al., 2022; Zhang et al., 2022; Zou et al., 2022). VMSFs have uniform and ordered arrays of nanochannels with adjustable diameters (2–11.8 nm, usually 2–3 nm), nanoscale film thicknesses (commonly 30–290 nm), and high porosity (∼75000 pore/μm (Henry and Sisler, 1984)) (Yan and Su, 2016; Zhou et al., 2020; Walcarius, 2021). In a general solution environment, the protonation of the silanol groups (p*K*_a_ 2–3) on the walls of VMSF nanochannels makes the surfaces negatively charged (Nasir et al., 2018; Wei et al., 2022). The nanochannel space presents significant charge-selective permeability, which can effectively enrich positively charged substances and repel negatively charged substances (Gong et al., 2022b; Gong et al., 2022c). In addition, negatively charged substances cannot reach the electrode surface due to electrostatic repulsion. Thus, the modified electrode can eliminate the interference of common redox small molecules (such as ascorbic acid-AA, uric acid-UA, etc.) in sample matrix (Zhou et al., 2018). In addition, the ultra-small nanochannels of VMSF can hinder the entry of substances larger than the pore size, which endows the VMSF modified electrode with good antifouling ability.

In this work, a three-dimensional, highly sensitive electrochemical detection platform was constructed by modifying VMSF on 3DG electrode, which can realize sensitive detection of TCP and indirect detection of PCZ in environmental and food samples. The 3DG electrode was pre-activated by a simple two-step electrochemical polarization process to introduce abundant active edge planes and oxygenous functional groups, which endow the pre-activated 3DG (p-3DG) with a large electroactive area resulted from the improved wettability and high electrocatalytic activity. The increased oxygen-containing functional groups on the surface of p-3DG also facilitate stable binding of VMSF with no need of additional adhesive layer. VMSF can be easily grow on p-3DG using an electrochemical assisted self-assembly (EASA) method. The obtained VMSF/p-3DG electrode combines the high electroactive area and good catalytic activity of p-3DG as well as the enrichment effect of VMSF on analytes, and the anti-fouling/anti-interference abilities. The fabricated VMSF/p-3DG sensor exhibits high electrochemical response towards TCP. Sensitive detection of TCP and prochloraz in complex samples was established.

## Materials and methods

### Chemicals and materials

Tetraethyl orthosilicate (TEOS, 98%), 2,4,6-trichlorophenol (TCP), 1-butyl-3-methylimidazolium ammonium hexafluorophosphate (BMIMPF_6_), disodium hydrogen phosphate heptahydrate (Na_2_HPO_4_·7H_2_O), sodium dihydrogen phosphate (NaH_2_PO_4_), potassium hydrogen phthalate (KHP), potassium ferricyanide (K_3_Fe(CN)_6_), and potassium ferrocyanide (K_4_[Fe(CN)_6_] were purchased from Shanghai Aladdin Reagent Co., Ltd (Shanghai, China). Hydrochloric acid (HCl) was purchased from Shuanglin Chemical Reagent Co., Ltd (Hangzhou, China). Cetyltrimethylammonium bromide (CTAB) and acetonitrile (99.9%) were obtained from Macklin Biochemical Technology Co., Ltd (Shanghai, China). All reagents and chemicals were used directly without further purification. Ultrapure water (18.2 MΩ cm) was prepared by Mill-Q system (Millipore Corporation) and used throughout the work. Phosphate buffer solution (PBS, 0.1 M) was prepared using Na_2_HPO_4_ and NaH_2_PO_4_. The stock solution of TCP was prepared by dissolving TCP in ethanol (10 mM) followed by serial dilution with PBS.

### Measurements and instrumentation

Scanning electron microscopy (SEM) measurement was performed on a SU8100 scanning electron microscope (Hitachi, Japan) with an accelerating voltage of 10 kV. All electrochemical tests including cyclic voltammetry (CV) and differential pulse voltammetry (DPV) were performed on a PGSTAT302N Autolab electrochemical workstation (Metrohm, Switzerland). The measurement adopts a conventional three-electrode system, with 3DG, p-3DG or VMSF/p-3DG as the working electrode (electrode size 0.5 cm × 0.5 cm), and platinum wire or platinum sheet as the counter electrode. The reference electrode for aqueous electrochemical measurements was an Ag/AgCl (saturated KCl solution) electrode. The Ag/Ag^+^ (10 mM Ag^+^/acetonitrile solution) was employed as the reference electrode in non-aqueous electrochemical experiments. In the DPV measurement, the step potential was 5 mV, the pulse amplitude was 25 mV, the pulse duration was 0.05 s, and the time interval was 0.2 s. Gas chromatography (GC) analysis was performed on a gas chromatographic instrument with an electron capture detector-ECD (Thermo, United States; quartz capillary column: 30 m × 0.32 mm × 0.25 μm; carrier gas: high-purityN_2_; flow rate: 2.5 ml/min; the inlet temperature: 240°C; the detector temperature: 300°C; the injection volume: 1 μL; Heating program: the initial temperature of 70°C, the final temperature of 245°C, heating rate of 40°C/min).

### Preparation of 3DG electrodes

According to the previous report, 3DG was synthesized by chemical vapor deposition (CVD) using nickel foam as the template (Zhu et al., 2022). The template-containing 3DG was subsequently soaked in HCl (3 M) at 80°C to remove Ni elements. To prepare 3DG electrodes, 3DG foam was cut into small pieces (0.5 cm × 0.5 cm) and then fixed onto the glass slide using silicone. Then, conductive silver was used to connect 3DG and the copper wire. Finally, the connection between the conductive silver glue and the copper wire was sealed with silicone.

### Preparation of p-3DG electrodes

Electrochemical polarization of 3DG electrodes was performed using an anodic oxidation in an organic phase followed by a cathodic reduction in an aqueous phase (Zhou et al., 2022c). The electrolyte for anodization was an acetonitrile solution containing the ionic liquid BMIMPF_6_ (10%, V/V). Specifically, a constant voltage of +5 V was applied to the 3DG electrode for 100 s. Subsequently, cathodic reduction was performed by applying a constant voltage of -1.0 V to the electrodes in PBS buffer (0.1 M, pH 6.0) for 300 s. The resulting electrode was named as p-3DG electrode.

### Preparation of VMSF/p-3DG electrodes

VMSF was grown on p-3DG electrodes using an EASA method (Walcarius et al., 2007). The precursor solution for VMSF growing was firstly prepared. Briefly, CTAB (1.585 g) and TEOS (2.833g) were added to the mixture of ethanol (20 ml) and NaNO_3_ solution (20 ml, 0.1 M), and the obtained solution (adjusted pH to be 3 with HCl) was stirred for 2.5 h. To grow VMSF, p-3DG was immersed in the precursor solution and a constant current of −350 μA was applied for 10 s. Afterwards, the electrode was immediately taken out and thoroughly washed with ultrapure water. Then, Scotch tape was used to clean the surface several times to remove possible agglomerates of silica. The obtained electrode was aged at 80°C overnight to obtain an electrode with micelle-blocked nanochannels (SM@VMSF/p-3DG). The SM@VMSF/p-3DG electrode was immersed in a hydrochloric acid/ethanol solution and stirred for 5 min to remove the inner micelles. Finally, the obtained was denoted as VMSF/p-3DG electrode, which contains open nanochannel array.

### Electrochemical detection of TCP and prochloraz

PBS (0.1 M, pH 6.0) was employed as the electrolyte for detection of TCP. DPV curves were measured on VMSF/p-3DG electrode in the presence of different concentrations of TCP after 20 min of agitation enrichment. For real sample analysis, environmental water samples were taken from a pond in Zhejiang Sci-Tech University (Hangzhou, China). The pond water was firstly filtered with a 0.22 μm filter film (nylon), and then diluted by a factor of 10 with PBS. The electrochemical determination of TCP was performed using a standard addition method. The green cabbage leaves and orange peels were purchased from the local supermarket (Hangzhou, China) and the samples were pretreated according to NY/T 1456–2007 (China). Finally, prochloraz and pyridinium hydrochloride were added to the obtained sample, and TCP was produced by hydrolysis of prochloraz (Li et al., 2010). To examine the reliability of the developed method, GC was used as the standard control method.

## Results and discussion

### Preparation and characterization of p-3DG

**FIGURE 1:**
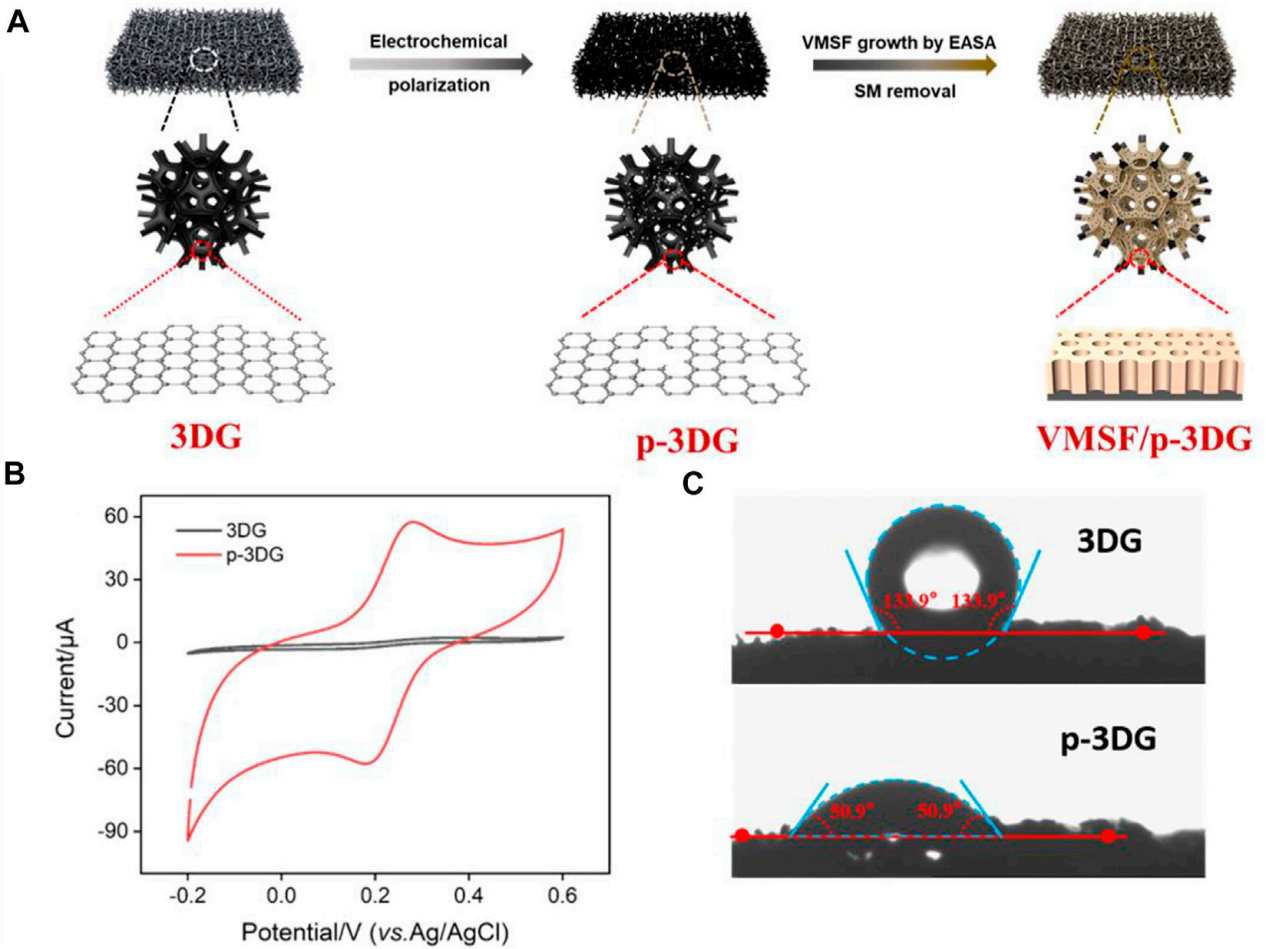
**(A)** Schematic illustration for the preparation of VMSF/p-3DG. **(B)** CV curves obtained at 3DG and p-3DG electrodes in Fe(CN)_6_^3−^ solution (0.5 mM) containing 0.1 M KCl. **(C)** Contact angle images of 3DG and p-3DG.

3DG can effectively overcomes the stacking and agglomeration problems of 2D graphene sheets while maintaining the excellent electron transport properties of graphene. Owing to continuous and seamless macroporous monolith structure, 3DG has a large surface area, high stability, and facilitated mass transfer of analytes (Chen et al., 2011; Liu et al., 2020). However, the high hydrophobicity and defect-free structure of 3DG hinder its electrocatalytic performance. In addition, direct growth of VMSF on its surface is difficult. As illustrated in Figure 1A, electrochemical polarization method is adopted as a simple and green method to pre-activate 3DG, which is followed by the growth of VMSF on the obtained p-3DG electrode. Electrochemical polarization includes anodic oxidation at high potential and cathodic reduction at low potential. To avoid the destroy on the structure of 3DG resulting from the large amount of gas generated by the decomposition of water, anodic oxidation is carried out in ionic liquid/acetonitrile. On the other hand, the cathodic reduction is then carried out in an aqueous phase. This electrochemical pre-activation can introduce ionic liquid and oxygen-containing groups on p-3DG, thereby improving its hydrophilicity and electrocatalytic performance (Zhou et al., 2022b). In addition, stable binding of VMSF on the surface of p-3DG is expected through the reaction between oxygen-containing groups (e.g., -OH group) and silanol groups.

The effect of electrochemical pre-activation on the properties of 3DG are characterized by electrochemical method and contact angle measurement. Figure 1B shows the cyclic voltammetry (CV) curves obtained on 3DG and p-3DG in standard redox probe Fe(CN)_6_^3−^. As shown, the redox peak current on p-3DG is significantly larger than that on 3DG. In addition, the charging current of p-3DG also became significantly larger. This is attributed to the increased electroactive area and improved hydrophilicity of p-3DG. Figure 1C shows the contact angle images of 3DG and p-3DG. The contact angle of 3DG is 133.9°, indicating its high hydrophobicity. In contrast, p-3DG has a contact angle of 50.9°, demonstrating a significantly improved hydrophilicity.

**FIGURE 2:**
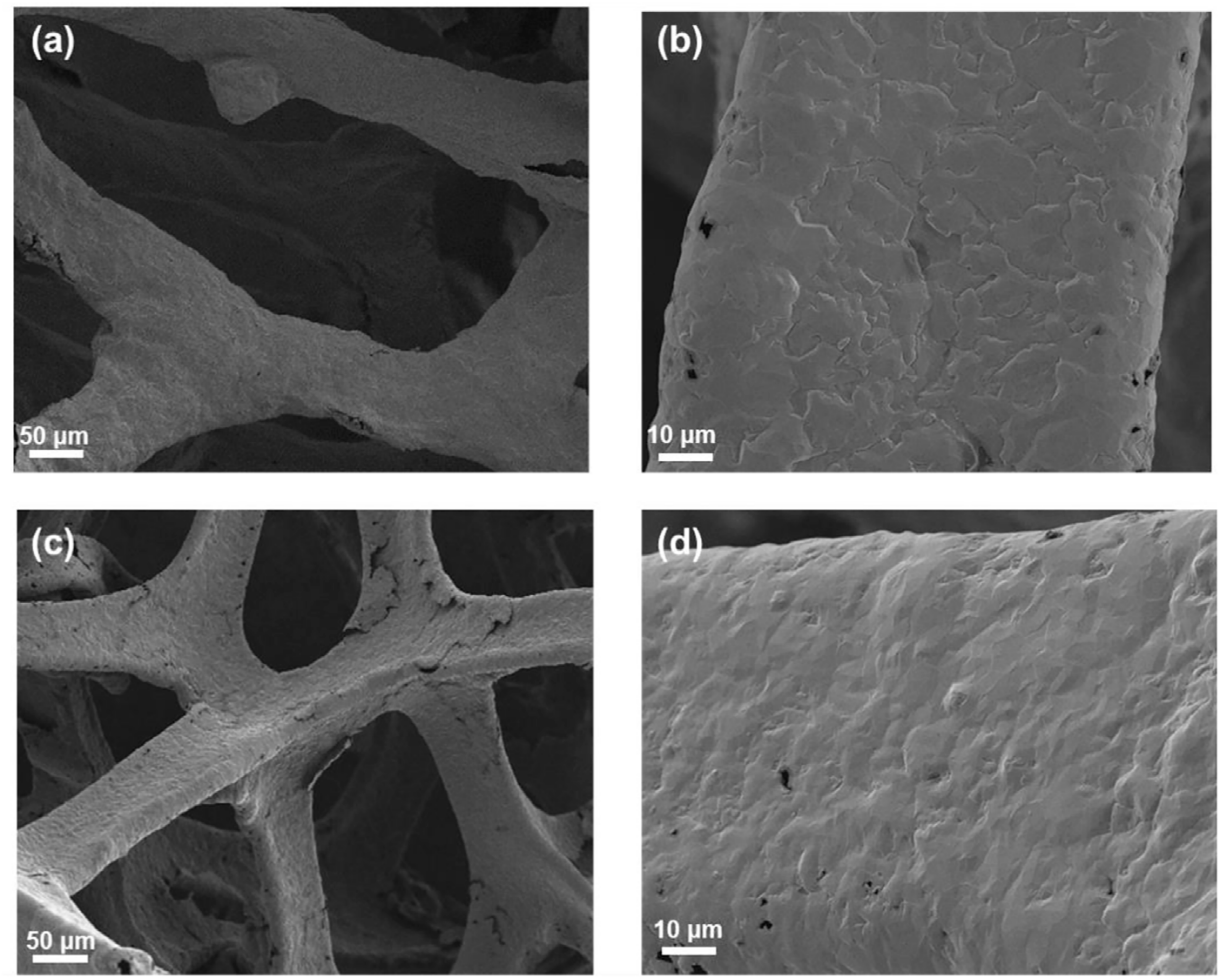
SEM images of 3DG **(A,B)** and p-3DG **(C,D)** at different magnification.

The morphologies of 3DG and p-3DG were characterized by scanning electron microscopy (SEM). Figure 2 shows the SEM images of 3DG (a and c) and p-3DG (b and d) under different magnifications. As seen, 3DG exhibits a macroporous graphene network structure, which will facilitate the mass transfer of analyte (Figure 2A). SEM image under high magnification clear reveals the wrinkle structure of graphene (Figure 2C). In case of p-3DG, the framework structure of 3DG remains (Figure 2B). However, some defect structures could be found on its surface resulting from the electrochemical etching in electrochemical polarization (Figure 2D).

### Stable binding of VMSF on p-3DG

**FIGURE 3:**
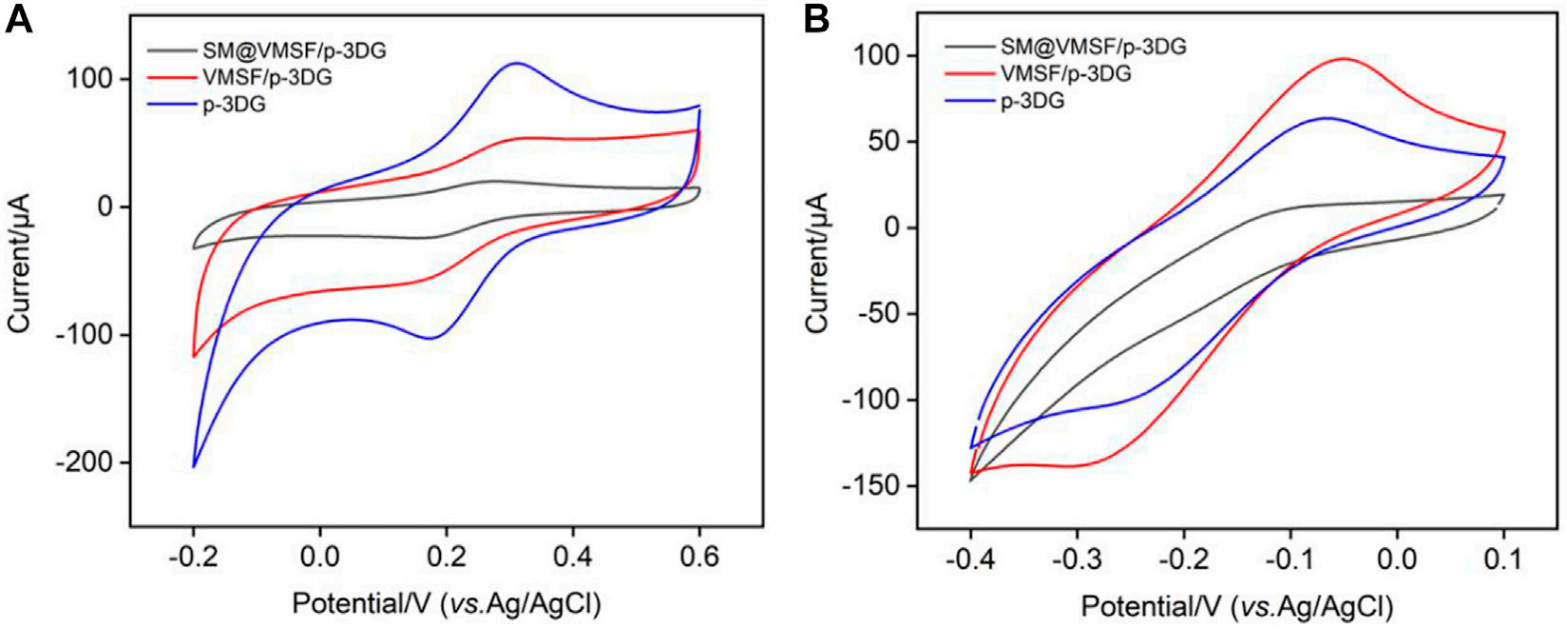
**(A)** CV curves obtained at different electrodes in Fe(CN)_6_^3−^ (0.5 mM) solution in potassium hydrogen phthalate (KHP, 0.05 M). **(B)** CV curves obtained at different electrodes in Ru(NH_3_)_6_^3+^ (0.5 mM) in PBS (0.01 M).

As illustrated in Figure 1, VMSF/p-3DG electrode is fabricated by rapid integration of VMSF on the surface of p-3DG using the electrochemical-assisted self-assembly (EASA) method (Walcarius et al., 2007; Xi et al., 2019). The integrity and charge-based permeation selectivity of the grown VMSF were characterized using standard electrochemical probes with opposite charges). As shown in Figure 3, electrochemical signals of SM@VMSF/p-3DG in both negatively charged Fe(CN)_6_^3−^ and positively charged Ru(NH_3_)_6_^3+^ are very low because SM blocks nanochannels, which further hinders the diffuse of redox probes to the electrode surface. After removal of SM, the CV signal of VMSF/p-3DG electrode is recovered in both solutions. The current signals obtained on SM@VMSF/p-3DG are very low compared to that on p-3DG or VMSF/3DG, however, this phenomenon is still different from the almost undetectable electrochemical signals on 2D planar electrodes. This might be due to the unique macroporous monolithic structure and high electronic conductivity of 3D graphene. Although a large amount of planar extension of p-3DG can grow VMSF, a small number of cross-sectionals sections or defects can still conduct electron transfer with the electrochemical probe, resulting in a low electrochemical signal. Compared with p-3DG electrode, the peak currents of VMSF/p-3DG electrode decrease in Fe(CN)_6_^3−^ solution, while the peak currents enhanced in Ru(NH_3_)_6_^3+^ solution. This is attributed to the charge-selective permeation of VMSF nanochannels. The deprotonation of silanols (p*K*_a_ 2–3) on the VMSF surface generates negative charges of nanochannels that repel negatively charged probes while attracting positively charged probes.

**FIGURE 4:**
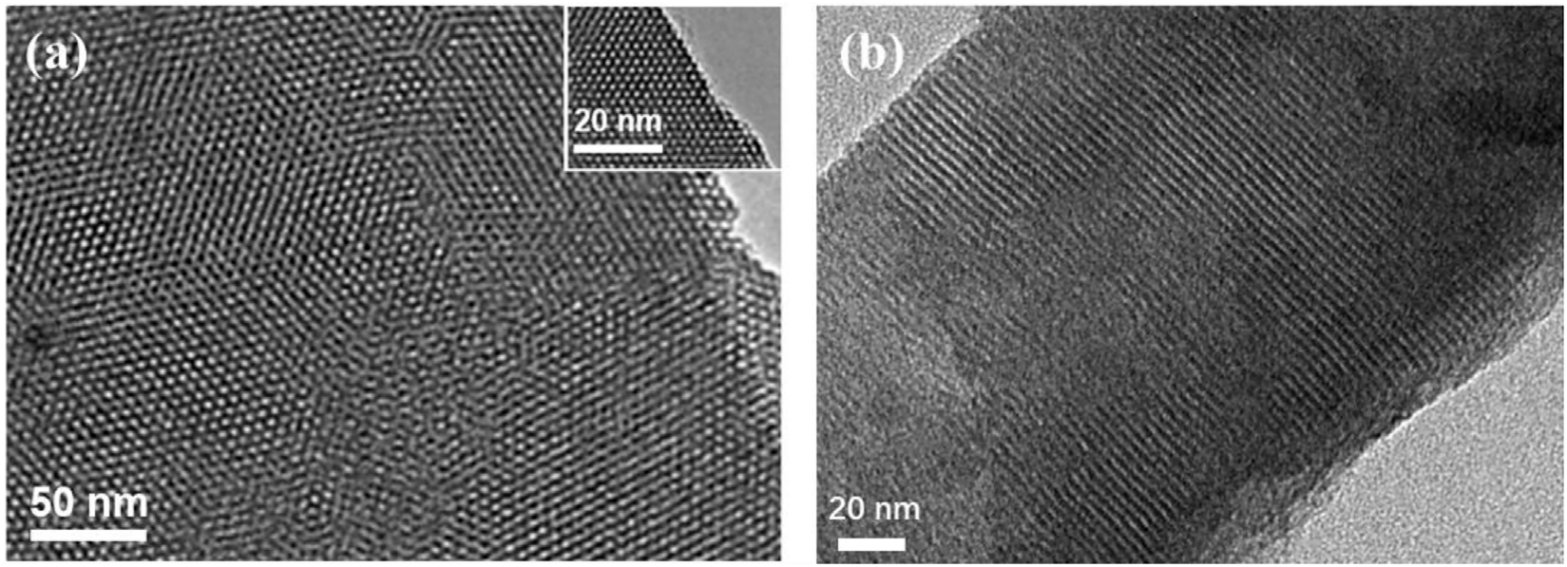
Top-view **(A)** and cross-sectional **(B)** TEM images of VMSF at different magnifications.

Transmission electron microscopy (TEM) is used to characterize the morphology of VMSF grown on p-3DG. Figure 4 displays the top-view and cross-sectional TEM images of nanochannel structure of VMSF. As shown, VMSF has a well-ordered structure with uniform pore size. The diameter of nanopore is between 2 and 3 nm (Figure 4A). The cross-sectional view reveals the parallel nanochannel structure (Figure 4B).

### Enrichment effect of VMSF nanochannel on TCP

**FIGURE 5:**
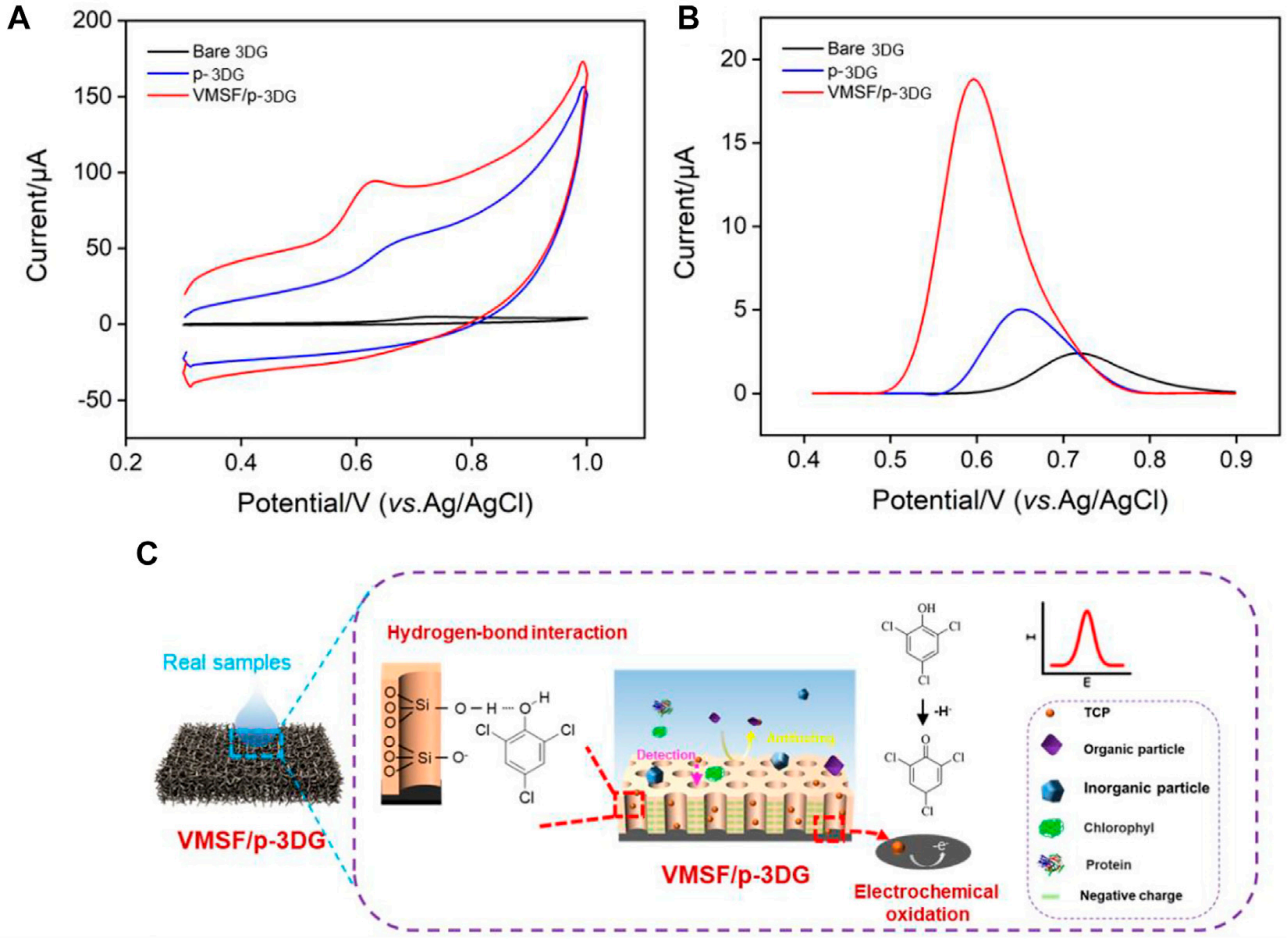
CV **(A)** and DPV **(B)** curves obtained at different electrodes in TCP solution (15 μM). **(C)** The illustration for the enrichment of TCP by VMSF nanochannel and the following electrochemical detection.

To verify the feasibility of the determination of TCP with VMSF/p-3DG electrode, the electrochemical responses of different electrodes towards TCP were compared. Figure 5A demonstrates CV curves obtained on 3DG, p-3DG and VMSF/p-3DG electrodes in TCP solution. As seen, the electrochemical reaction of TCP is irreversible with remarkable oxidation peak. Compared with 3DG electrode, p-3DG shows a significantly enhanced electrochemical peak current. The significant increase in the charging current also demonstrates the increase in the active area of the electrode. In addition, VMSF/p-3DG electrode exhibits the highest oxidation peak current, indicating the signal amplification effect of VMSF nanochannels. Figure 5B displays the corresponding DPV curves obtained on different electrodes. The p-3DG electrode exhibits lower oxidation potential compared with 3DG, proving the electrocatalytic performance of p-3DG. This is attributed to the oxygen-containing functional groups and the defect sites of graphene introduced in the electrochemical polarization process, which can be used as active sites to improve the electrocatalytic performance of the electrode. For VMSF/p-3DG electrode, lower oxidation potential and higher peak current are obtained, indicating that VMSF nanochannel has significant enrichment effect on TCP. Figure 5C demonstrates the possible mechanism of the nanochannel-based signal amplification and the following electrochemical detection. Briefly, TCP interacts with silanol groups on the surface of VMSF through hydrogen bonding (Xuan et al., 2021; Yan et al., 2021), thereby realizing significant enrichment of TCP.

**FIGURE 6:**
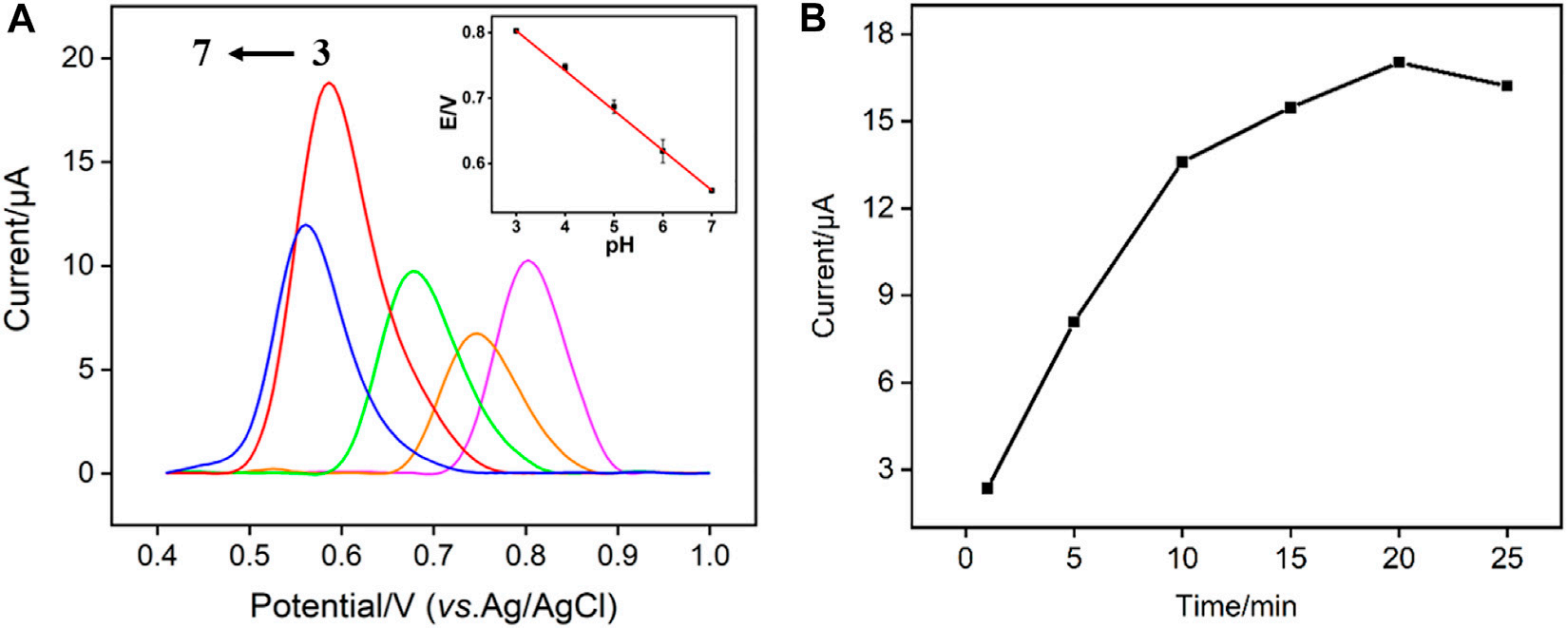
**(A)** DPV curves of TCP (15 μM) obtained on VMSF/p-3DG electrode at different pH. Inset is the dependence of oxidation peak potential (E) and on the pH value. **(B)** DPV oxidation peak current obtained using different enrichment time.

To achieve the highest detection sensitivity, the effect of detection pH and enrichment time on the electrochemical response of TCP are studied. As shown in Figure 6A, the VMSF/p-3DG electrode has the highest peak current at pH 6. At the same time, the oxidation potential (*E*) decreases linearly with the increase of pH (E = −0.06098pH + 0.9860, *R* ^2^ = 0.999), indicating H^+^ ion is involved in the electrochemical reaction of TCP on the electrode (as illustrated in Figure 5C). A slope close to 0.059 indicates that the same number of electrons and protons are involved in the electrochemical reaction. As shown in Figure 6B, the peak current of TCP increases with the enrichment time and reaches a maximum at 20 min.

### Sensitive detection of TCP using VMSF/p-3DG electrode

**FIGURE 7:**
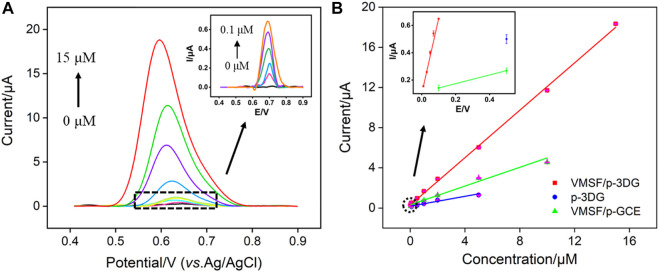
**(A)** DPV curves obtained on VMSF/p-3DG electrode at different concentration of TCP. Inset is the magnified view of the DPV curves in the low-concentration region. **(B)** The calibration curve for detection of TCP using different electrodes. Inset is the magnified view in the low-concentration region.

The detection of TCP using VMSF/p-3DG electrode is performed using DPV under the optimized conditions. Figure 7A displays the DPV curves obtained on VMSF/p-3DG electrode in presence of different concentrations of TCP. As seen, the DPV oxidation peak current increases with the increase of TCP concentration. When the concentration of TCP is in the range of 10 nM–0.1 μM and 0.1–15 μM, a linear relationship between the DPV oxidation peak current (*I*) and TCP concentration (*C*) is observed (Figure 7B, *I* = 5.517*C* + 0.09787, *R* ^2^ = 0.998; *I* = 1.159C + 0.4949, *R* ^2^ = 0.998, respectively). The limit of detection (LOD) calculated based on the three signal-to-noise (S/N = 3) is 2.4 nM. To verify the signal amplification effect of VMSF, the detection of TCP using p-3DG is also investigated. As shown in Figure 7B, the p-3DG electrode can linearly detect TCP in the range of 50 nM–5 μM (*I* = 0.6020*C* + 0.1836, *R* ^2^ = 0.9903) with a LOD of 28 nM. Furthermore, VMSF-based 2D electrode is also prepared through VMSF growth on electrochemical pre-treated glassy carbon electrode (VMSF/p-GCE) to verify the advantages of 3D electrodes. The linear detection range of TCP obtained on VMSF/p-GCE is 0.5–10 μM (*I* = 0.1218*C* + 0.1385, *R* ^2^ = 0.9859, Figure 6B) and the LOD is 0.49 μM. Therefore, the VMSF/p-3DG electrode has the widest linear range and the lowest LOD, which is attributed to the combination of the high active area of the 3D graphene electrode and the signal amplification of VMSF.

### Selectivity, anti-fouling, reusability abilities of VMSF/p-3DG electrode and real sample analysis

**FIGURE 8:**
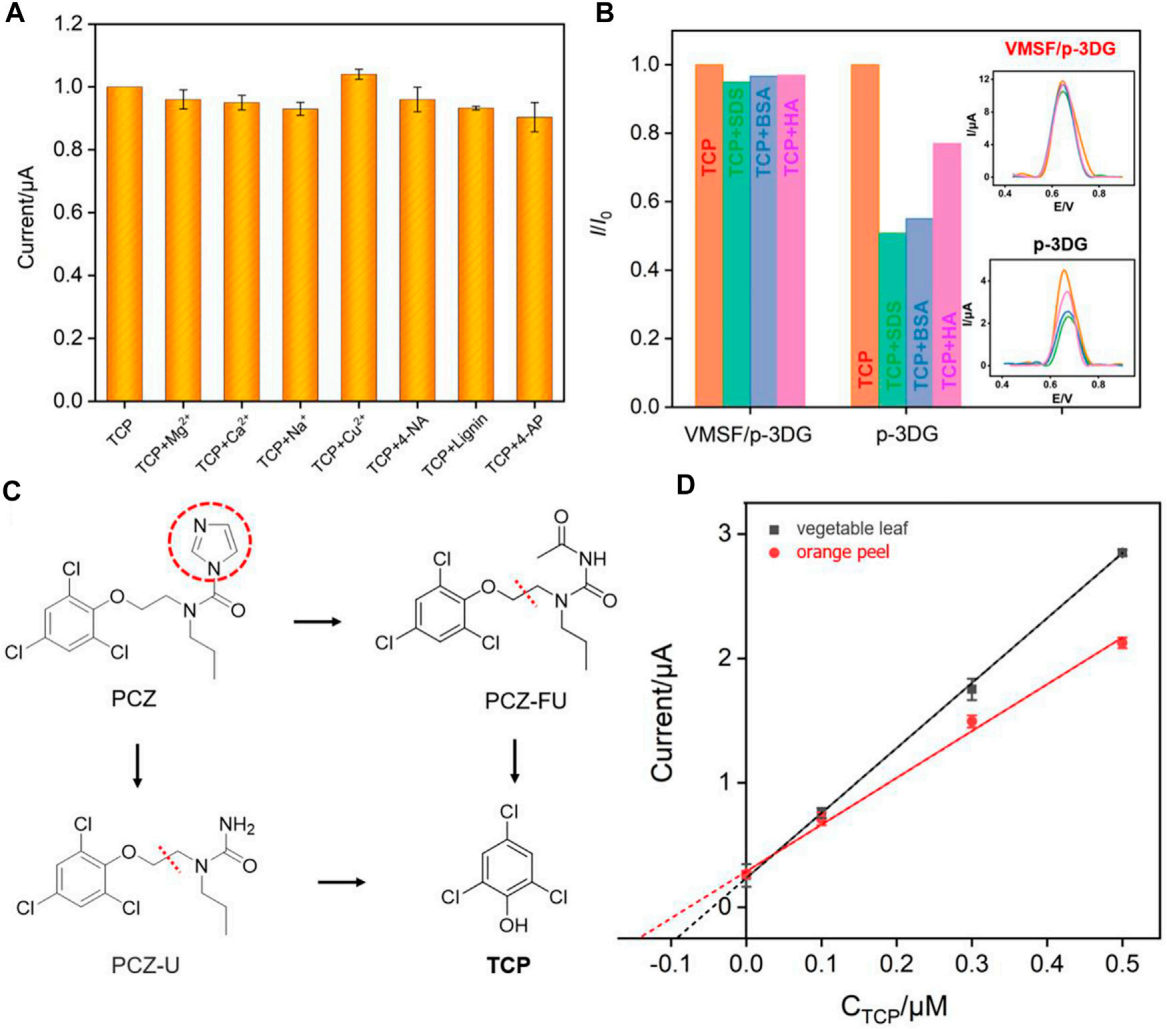
**(A)** The current ratio (*I*/*I*_0_) obtained from VMSF/p-3DG for detection of TCP (2 μM) in the absence (*I*_0_) and presence (*I*) of 50-fold of other added species. **(B)** The current ratio (*I*/*I*_0_) obtained from VMSF/p-3DG or p-3DG for detection of TCP (2 μM) in the absence (*I*_0_) and presence (*I*) of added species. Inset are the corresponding DPV curves. **(C)** The illustration for the metabolic process of PCZ. **(D)** The calibration curve for detection of TCP in the extracts of orange leaf and orange peel using extrapolation in standard addition method.

The selectivity of VMSF/p-3D-G electrode is investigated. Some possible interfering substances including metal ions (Mg^2+^, Ca^2+^, Na^+^ and Cu^2+^), organic redox small molecules (p-nitroaniline 4-NA and p-aminophenol, 4-AP), and the biopolymer lignin are chosen to investigate the effect on the electrochemical signal of TCP. As shown in Figure 8A, these substances almost have no effect on the detection of TCP although each concentration is 50 times higher than that of TCP, indicating that VMSF/p-3DG electrode has good selectivity. Furthermore, the anti-smudge ability of VMSF/p-3DG electrode is also studied. Macromolecules, that are commonly found in complex sample matrices, including bovine serum albumin (BSA), sodium dodecyl sulfonate (SDS), and humic acid (HA), are added to TCP solution. As shown in Figure 8B, the response of TCP on p-3DG electrode significantly decreased, indicating that these substances are easy to contaminate the electrode and reduce the accuracy of electrode detection. In contrast, the DPV peak currents obtained VMSF/p-3DG electrode does not change significantly when these macromolecules are present, demonstrating excellent anti-fouling performance. This is attributed to the size exclusion effect of the ultrasmall nanochannels of VMSF. Since these macromolecules cannot enter the nanochannels, they cannot contaminate the electrode surface. The regeneration and reusability of the fabricated VMSF/p-3DG sensor are investigated. The electrode can be easily regenerated by soaking in hydrochloric acid-ethanol solution (0.1 M) for 3 min. As revealed in Supplementary Figure S1 (in supporting information, SI), electrodes can be easily regenerated and the regenerated electrodes have no signals of TCP in the electrolyte. On the other hand, the signals of TCP on the regenerated VMSF/p-3DG sensor have no significant change. Thus, the developed VMSF/p-3DG sensor show great potential in direct electroanalysis of complex samples. Thus, the environmental water samples are directly analyzed by standard addition method using VMSF/p-3DG sensor. As shown in Supplementary Table S1, the detection has satisfactory recoveries ranging from 95.0–104% with a relative standard deviation (RSD) less than 3.5%, indicating high detection accuracy.

### Indirect detection of PCZ using VMSF/p-3DG electrode

Prochloraz (PCZ) itself has no electrochemical response. The metabolic process of PCZ is shown in Figure 8C. After the imidazole ring of PCZ is cleaved, two intermediate products including N-formyl-N-propyl-N-[2-(2,4,6-tri-chlorophenoxy)ethyl]urea (PCZ-FU) and N-propyl-N-[2-(2,4,6-trichlorophenoxy)ethyl]urea (PCZ-U) are formed, which are further degraded to form TCP (Li et al., 2010). In this process, PCZ is hydrolyzed to produce an equivalent amount of TCP. According to the molecular weight (Mw) of the two substances (376.5 for PCZ and 197.5 for TCP), the mass or content of TCP multiplies by a factor of 1.906 is the mass or content of PCZ. Thus, the strategy for the indirect detection of prochloraz is generally based on the direct detection of TCP. Generally, pyridine hydrochloride is added into samples containing PCZ to convert prochloraz and its metabolic intermediates into TCP. Then, PCZ is indirectly determined by determining the content of TCP (Debrauwer et al., 2001; Li et al., 2010). To further verify the application of the developed sensor, the detection of PCZ in the extracts of vegetable (green cabbage) leaves and orange peel are investigated. The prochloraz standard solution is added into the extracts of vegetable leaves or orange peel and then converted into TCP by heating and refluxing with pyridine hydrochloride. Then, the content of PCZ in the original sample was determined by extrapolation. As shown in Figure 8D, the slope of the linear curve of TCP concentration is different in orange peel and vegetable leaves. This phenomenon is ascribed to the matrix effect in different real samples. The determined concentration of prochloraz in the green cabbage leaves or orange peel was 1.74 mg/ml and 2.73 mg/ml, respectively. When the samples were analyzed using GC method, the concentrations of PCZ were 1.68 mg/ml and 2.70 mg/ml, respectively. The close results indicated the reliability of the developed electrochemical method for the detection of PCZ.

## Conclusion

In summary, we have developed a 3D electrochemical sensing platform for sensitive detection of TCP in environmental samples and prochloraz in food samples by integrating VMSF on a 3D graphene (3DG) electrode with no need of additive adhesive layer. The electrochemically preactivated 3DG (p-3DG) electrode exhibits high electroactive area and excellent electrocatalytic performance. The rapid growth of VMSF on p-3DG is easily achieved by an electrochemically assisted self-assembly method. The nanochannels of VMSF could enrich TCP through hydrogen bonding, leading to remarkable signal amplification in detection. As a nanofilter, VMSF endows the VMSF/p-3DG electrode with excellent antifouling ability. Sensitive detection of TCP in lake water and indirect detection of prochloraz in vegetable leaves and orange peels were achieved. Compared with the existing TCP and prochloraz detection methods, the developed VMSF/p-3DG sensor has simple electrode structure and excellent sensing performance. Combined with the commercialization of 3DG and the functional groups or nanomaterials modification of VMSF nanochannels, the sensors constructed here can be extended for sensitive electrochemical analysis in the fields of medicine, biology, food, and environment.

## Data Availability

The original contributions presented in the study are included in the article/Supplementary Material, further inquiries can be directed to the corresponding authors.
