# Supplementary material for: Nanochannel array modified three-dimensional graphene electrode for sensitive electrochemical detection of 2,4,6-trichlorophenol and prochloraz
Source: Front Chem. 2022 Sep 7;10:954802. doi: 10.3389/fchem.2022.954802 (PMC9490055; doi:10.3389/fchem.2022.954802)
Supplement: Supplementary file 1 [file DataSheet1.PDF]

Table S1 Determination of TCP in pond water samples.

Sample	Spiked (μM)	Found (μM)	RSD (%, n=3)	Recovery (%)
pond water[a]	0.100	0.095	3.5	95.0
	1.00	1.04	2.7	104
	2.00	1.99	1.7	99.7

[a]Samples with added TCP were diluted by a factor of 10 using the electrolyte. The concentration of TCP was the added concentration after dilution.

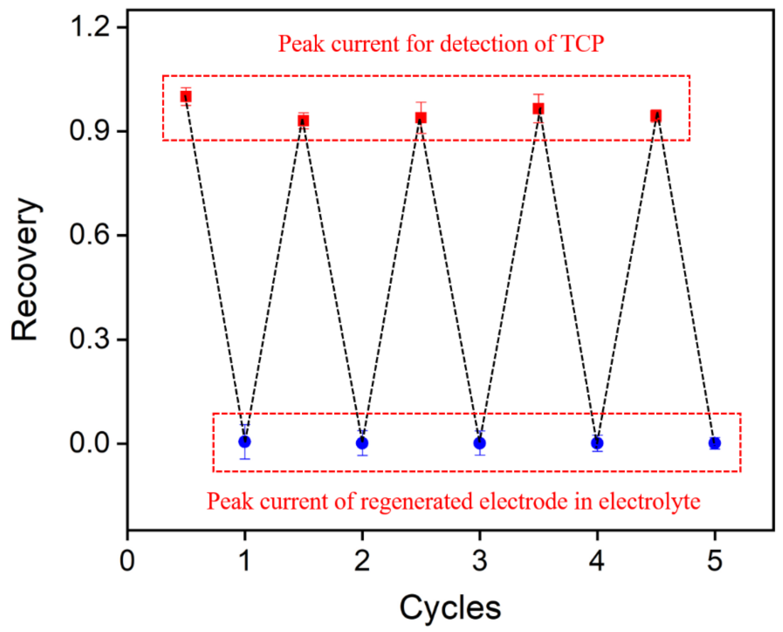

Figure S1 The reuse performance of VMSF/p-3DG. The first peak current is obtained using the original electrode towards TCP. Other peak cur-rents are obtained in electrolyte (bottom) or TCP solution (top) using the regenerated electrodes.
